# Dynamic Predictive Models of Cardiogenic Shock in STEMI: Focus on Interventional and Critical Care Phases

**DOI:** 10.3390/jcm14103503

**Published:** 2025-05-16

**Authors:** Elena Stamate, Anisia-Luiza Culea-Florescu, Mihaela Miron, Alin-Ionut Piraianu, Adrian George Dumitrascu, Iuliu Fulga, Ana Fulga, Octavian Stefan Patrascanu, Doriana Iancu, Octavian Catalin Ciobotaru, Oana Roxana Ciobotaru

**Affiliations:** 1 Department of Morphological and Functional Sciences, Faculty of Medicine and Pharmacy, “Dunarea de Jos” University of Galați, 35, Al. I. Cuza Street, 800216 Galați, Romania; elena.stamate@ugal.ro; 2 Department of Electronics and Telecommunications, “Dunarea de Jos” University of Galați, 800008 Galați, Romania; 3 Department of Computer Science and Information Technology, “Dunarea de Jos” University of Galați, 800008 Galați, Romania; mihaela.miron@ugal.ro; 4 Division of Hospital Internal Medicine, Department of Medicine, Mayo Clinic Florida, 4500 San Pablo Rd S, Jacksonville, FL 32224, USA; 5 Department of Medical, Faculty of Medicine and Pharmacy, “Dunarea de Jos” University of Galați, 35, Al. I. Cuza Street, 800216 Galați, Romania; iuliu.fulga@ugal.ro; 6 Department of Clinical Surgical, Faculty of Medicine and Pharmacy, “Dunarea de Jos” University of Galați, 35, Al. I. Cuza Street, 800216 Galați, Romania; ana.fulga@ugal.ro (A.F.); octavian.ciobotaru@ugal.ro (O.C.C.); 7 Faculty of Medicine and Pharmacy, “Dunarea de Jos” University of Galați, 35 AL Cuza St, 800010 Galati, Romania; octav974@gmail.com (O.S.P.);; 8 Department of Clinical Medical, Faculty of Medicine and Pharmacy, “Dunarea de Jos” University of Galați, 35, Al. I. Cuza Street, 800216 Galați, Romania; roxana.ciobotaru@ugal.ro

**Keywords:** STEMI, cardiogenic shock, machine learning, dynamic risk, post-revascularization monitoring, cardiac intensive care units, predictive models

## Abstract

**Background**: While early risk stratification in STEMI is essential, the threat of cardiogenic shock (CS) persists after revascularization due to reperfusion injury and evolving instability. However, risk prediction in later phases—after revascularization—is less explored, despite its importance in guiding intensive care decisions. This study evaluates machine learning (ML) models for dynamic risk assessment in interventional cardiology and cardiac intensive care unit (CICU) phases, where timely detection of deterioration can guide treatment escalation. **Methods**: We retrospectively analyzed clinical and procedural data from 158 patients diagnosed with STEMI complicated by cardiogenic shock, treated between 2019 and 2022 at the Cardiology Department of the University Emergency Hospital of Bucharest, Romania. Machine learning models—Random Forest (RF), and Quadratic Discriminant Analysis (QDA)—were developed and tested specifically for the interventional cardiology and CICU phases. Model performance was evaluated using area under the receiver operating characteristic curve (ROC-AUC), accuracy (ACC), sensitivity, specificity, and F1-score. **Results**: In the interventional phase, RF and QDA achieved the highest accuracy, both reaching 87.50%. In the CICU, RF and QDA demonstrate the best performance, reaching ACCs of 0.843. QDA maintained consistent performance across phases. Relevant predictors included reperfusion strategy, TIMI flow before percutaneous coronary intervention (PCI), Killip class, creatinine, and Creatine Kinase Index (CKI)—all parameters routinely assessed in STEMI patients. These models effectively identified patients at risk for post-reperfusion complications and hemodynamic decline, supporting decisions regarding extended monitoring and ICU-level care. **Conclusions**: Predictive models implemented in advanced STEMI phases can contribute to dynamic, phase-specific risk reassessment and optimize CICU resource allocation. These findings support the integration of ML-based tools into post-PCI workflows, enabling earlier detection of clinical decline and more efficient deployment of intensive care resources. When combined with earlier-stage models, the inclusion of interventional and CICU phases forms a dynamic, end-to-end risk assessment framework. With further refinement, this system could be implemented as a mobile application to support clinical decisions throughout the STEMI care continuum.

## 1. Introduction

While early triage is essential in ST-elevation myocardial infarction (STEMI) complicated by cardiogenic shock (STEMI-CS), clinical deterioration can also occur after initial revascularization, particularly in the interventional and intensive care phases. Post-angioplasty complications such as reperfusion injury, progressive ventricular dysfunction, and delayed hemodynamic collapse often remain underrecognized [1,2,3]. For these reasons, risk assessment should not be a one-time event, but a continuous and dynamic process adapted to each care stage.

The SCAI SHOCK classification supports stage-based clinical decision-making, yet the implementation of predictive systems in later phases of care is still limited [4,5]. Patients often exhibit evolving hemodynamic instability in the hours or days following percutaneous coronary intervention (PCI), necessitating advanced tools to identify and monitor those at risk for delayed cardiogenic shock [1,6].

**Figure 1:**
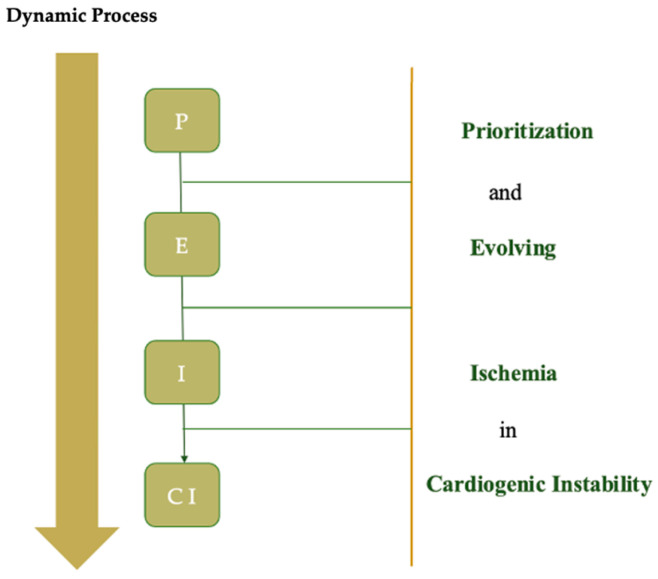
Dynamic process of progression to cardiogenic shock in STEMI patients.

**Figure 2:**
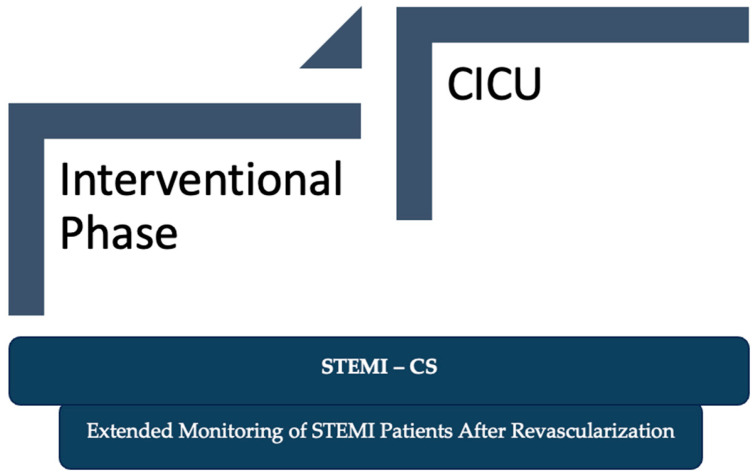
Dynamic surveillance of STEMI patients following PCI: From catheterization lab to CICU.

This article introduces the P-E-I-CI Model (Prioritization and Evolving Ischemia in Cardiogenic Instability), as illustrated in Figure 1, as a framework for machine learning-based dynamic risk assessment across interventional phase and cardiac intensive care units (CICU) or intensive care units (ICU) phases, as shown in Figure 2. The model is not a numerical score, but a methodology aimed at supporting ongoing risk evaluation through phase-specific prediction.

Artificial intelligence (AI) plays a key role in this strategy by enabling the processing of high-volume, multi-parametric data and improving risk prediction beyond conventional methods [7,8]. Prior studies highlight AI’s capacity to enhance diagnostic speed and therapeutic precision [9,10,11,12], improve outcomes [1,8,13,14], and reduce readmissions and complications [15,16,17,18]. Tailoring post-revascularization management to the evolving clinical profile may also reduce healthcare costs and improve quality of life [1,15,19,20,21,22].

However, critical knowledge gaps remain, including the absence of validated biomarkers for early detection, insufficient multicenter data, and the need for personalized, patient-centered prediction models [23,24,25,26,27,28]. Additionally, incorporating patient-reported outcomes (PROMs) and experience measures (PREMs) could provide a more complete view of long-term impact [17,29,30].

**Figure 3:**
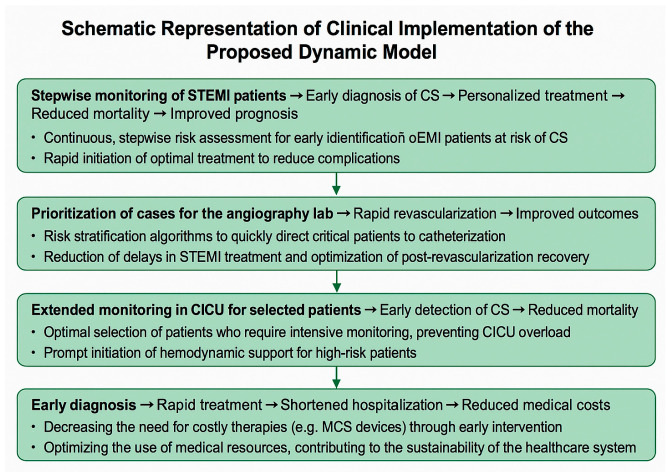
Schematic representation of clinical implementation of proposed dynamic model.

Thus, this study’s main objective is to improve clinical decision-making in STEMI-CS management by supporting the development of AI-assisted protocols aimed at early and personalized intervention. The added value of this research lies in extending risk monitoring for cardiogenic shock beyond the early phases previously addressed in the literature, into the post-revascularization stages; specifically, interventional cardiology and cardiac intensive care phases of care. The proposed model is dynamic and phase-specific, covering the entire continuum of care for STEMI patients—from initial medical contact, through diagnostic and interventional treatment, to intensive care monitoring. As illustrated in Figure 3, this model can be translated into a structured clinical care pathway, with a focus on early diagnosis, treatment prioritization, and optimal resource utilization.

Through stepwise risk monitoring, timely angiography, targeted CICU care, and early discharge planning, the model aims to reduce mortality and complications associated with STEMI-CS while alleviating the burden on the healthcare system. Built on the P-E-I-CI framework, this approach could evolve into a validated clinical score, fully integrable into decision-making algorithms, supporting efficient risk stratification and truly patient-centered care.

## 2. Materials and Methods

### 2.1. Dataset

**Figure 4:**
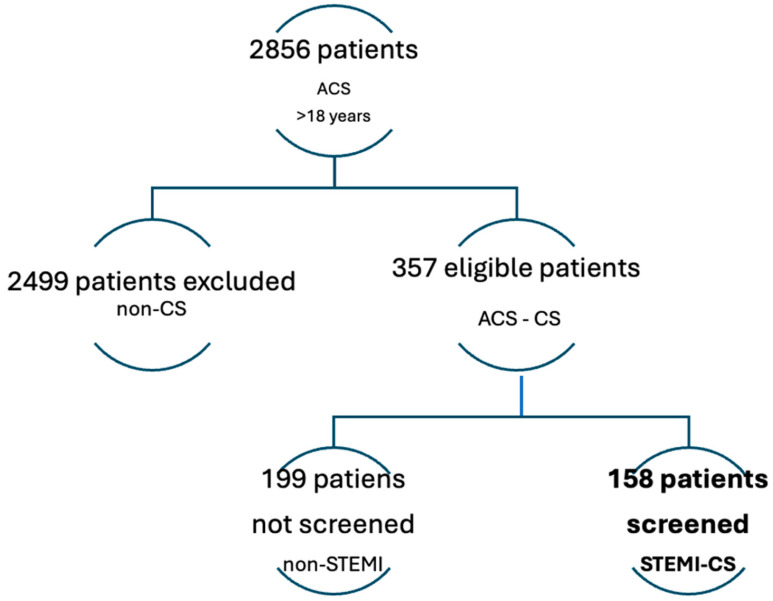
Flow chart of patient selection.

This retrospective observational study included data from 2856 adult patients (>18 years) admitted with acute coronary syndrome (ACS) [29] at the University Emergency Hospital of Bucharest between 2019 and 2022. Of these, 357 were diagnosed with cardiogenic shock (CS) as per contemporary clinical criteria [31]. After excluding cases not attributed to ST-elevation myocardial infarction (STEMI), 158 patients with confirmed STEMI-CS were selected for analysis, as shown in Figure 4.

The cohort was divided based on the timing of CS present on admission and CS post admission. This manuscript specifically focuses on the advanced stages of care—interventional cardiology and cardiac intensive care unit (CICU)—to identify predictors of adverse evolution in the post-revascularization setting and support phase-specific risk assessment through artificial intelligence–based models. The study adhered to the Declaration of Helsinki. Because all data were anonymized and analyzed retrospectively, informed consent was not required. Ethical approval was obtained according to institutional protocols.

Inclusion Criteria:

1. Age >18 years.
2. Acute coronary syndrome complicated by cardiogenic shock in patients admitted in the Cardiology Unit of the University Emergency Hospital of Bucharest.

Exclusion Criteria:

1. Patients with cardiogenic shock due to etiologies other than STEMI, such as non-ischemic cardiomyopathy (according to the ESC definition [32]), NSTEMI [33], unstable angina [33], pulmonary embolism [34], myocarditis [35], or infectious endocarditis [36].
2. Patients with a diagnosis of sepsis [37].
3. Medical records with missing hospitalization data.
4. Patients who requested to be discharged against medical advice.
5. Patients with end-stage liver disease.
6. Patients with coagulation disorders: patients with diagnosis of thrombophilia and patients with coagulopathy.
7. Patients with severe malnutrition.
8. Patients with other severe infections without a diagnosis of sepsis.
9. Patients receiving large-volume blood transfusions.
10. Patients with a diagnosis of active malignancy.

### 2.2. Analyzed Variable

We analyzed the medical records of the included patients, and we mapped the STEMI patient’s clinical course into two distinct phases: interventional cardiologist phase and cardiac intensive care unit/intensive cardiac unit phase.

The variable included in the analysis were:

- Demographic Data: Age, Sex.
- Clinical Parameters: Heart rate, Killip class (according to the definition [38]), time from symptom onset, need for vasopressor/inotropic support during hospitalization, need for mechanical ventilatory support during hospitalization.
- Cardiovascular Risk Factors: Diabetes mellitus (according to the definition [39]), hypertension (as defined by 2024 ESC Guidelines [40], active smoking at the time of admission, dyslipidemia: new diagnosis and history of dyslipidemia under treatment (based on the 2019 ESC definition [41].
- Laboratory Tests: Hemoglobin, leukocyte count, troponin, CKMB, Creatine Kinase–MB Isoenzyme (CKMB), Creatine Kinase Index (CKI), glucose, creatinine, potassium (K), sodium (Na), aspartate aminotransferase (AST), ALT (alanine aminotransferase), urea nitrogen (BUN), fibrinogen.
- Echocardiographic Data (Cardiology Phase): left ventricular ejection fraction (LVEF) (EF > 50%, EF 40–50%, EF < 40%), right ventricular (RV) dysfunction, left ventricular (LV) thrombosis, mitral regurgitation, LV aneurysm, mechanical complications and pericardial effusion.
- Electrocardiographic (ECG) Findings: ECG rhythm (sinus rhythm, atrial fibrillation, ventricular tachycardia, ventricular fibrillation, junctional rhythm), conduction abnormalities, QRS complex duration, ST elevation in aVR lead, ST elevation location on ECG, presence of Q Wave, reciprocal ST depression.
- Angiographic Findings: reperfusion type, the location of coronary artery occlusion, the number of affected epicardial coronary arteries, whether the patient underwent percutaneous coronary intervention (PCI), total number of drug-eluting stent (DES) implanted, atherosclerotic coronary artery disease, (PCI), pre-PCI TIMI.
- CICU specific parameters: the occurrence of cardiac arrest during hospitalization, events of resuscitation during hospitalization, ICU admission, the need for ventilatory support, the use of inotropic and vasopressor support, and the occurrence of mechanical complications.

The development of predictive models included clinical, biochemical, angiographic, and ICU-specific parameters considered to be relevant for assessing the risk of progression to cardiogenic shock in STEMI patients based on current medical knowledge. The Killip class at presentation was used as a validated clinical marker, being directly associated with patient prognosis and supporting a rapid decision-making regarding coronary intervention or hemodynamic support. Biochemical parameters such as creatinine, CKI, potassium, and hemoglobin were integrated to reflect the metabolic status and the degree of cardiac and renal impairment. Angiographic data, including the type of reperfusion, TIMI flow grade, and coronary occlusion site, were used to assess the severity of ischemia and the effectiveness of coronary intervention. Additionally, ICU-specific parameters, such as the need for ventilatory or inotropic support and the type of mechanical circulatory support used, were included to reflect the degree of hemodynamic instability and recovery potential, thus contributing to more accurate prediction and tailored therapeutic strategies.

### 2.3. Data Preprocessing

The development of predictive models in these advanced stages incorporated variables that reflect not only anatomical and procedural factors but also the hemodynamic response following revascularization.

- Interventional Cardiologist Phase: Demographic data, clinical parameters, cardiovascular risk factors, laboratory tests, and angiographic findings. ECG and echocardiographic data were excluded from this phase, as they may reflect the anatomical location of the culprit lesion and could introduce collinearity with angiographic findings, potentially biasing model interpretability.
- CICU Phase: Demographic data, cardiovascular risk factors, clinical and biological parameters, ECG findings, echocardiographic findings, and all previously mentioned angiographic parameters, along with additional data such as the occurrence of cardiac arrest during hospitalization, events of resuscitation during hospitalization, ICU admission, the need for ventilatory support, and the use of inotropic and vasopressor support.

Variable selection was based on clinical relevance, routine availability during advanced STEMI care, and their established association with cardiogenic shock prognosis.

We prioritized parameters that are easy to obtain in clinical practice and routinely documented in cardiology and intensive care units. This pragmatic approach aimed to maximize real-world applicability while enhancing early recognition of adverse events in the post-angioplasty setting. These advanced-phase models serve as the foundation for the P-E-I-CI framework proposed in this study. The predictive models were designed for binary classification: identifying high-risk patients for CS deterioration versus those with stable evolution post-PCI.

### 2.4. Statistical Analysis

Statistical analysis was performed on data collected from a patient cohort evaluated across interventional cardiology phase, and cardiac intensive care unit/intensive unit phase. Based on the general theoretical framework, this study introduces an innovative approach by developing phase-specific predictive models aimed at the early dynamic risk assessment detection of clinical deterioration in patients with STEMI in post-revascularization phase, supporting timely treatment escalation. The primary objective was to investigate how clinical and laboratory tests, collected at distinct phase of the care continuum, could contribute to the earlier identification of CS risk.

This stratified approach enables a comprehensive view of disease progression and facilitates timely clinical decision-making. A critical component of this process was identifying the most relevant parameters that influence CS risk at each phase of care, enhancing both risk stratification and individualized patient management. In the initial phase, Random Forest (RF) was employed for feature selection, owing to its advantages in handling high-dimensional medical data and its ability to model complex, non-linear interactions without requiring prior assumptions. RF’s built-in feature importance mechanism allowed for the identification and ranking of the top seven predictors most informative for CS risk, thus reducing dimensionality while preserving model performance. The predictive strength of the model was assessed using accuracy, F1-score, and Matthews correlation coefficient, with 95% confidence intervals calculated to ensure robustness in clinical application. To further interpret the role of each selected variable, logistic regression (LR) was applied, offering transparency and inferential value. Multicollinearity was assessed using the Variance Inflation Factor, ensuring model stability and interpretability. Finally, McNemar’s test was employed to statistically compare the binary predictions of RF and LR, particularly evaluating the effect of including interaction terms in LR or model selection strategy. While not assessing overall accuracy, this test confirmed whether the two models made significantly different predictions on the same cases, providing additional insight into their comparative diagnostic utility.

In many real-world applications, particularly in fields like medicine, model calibration is as crucial as its ability to discriminate between classes. To enhance the evaluation of model performance, we included the Brier score, a metric that assesses calibration by comparing predicted probabilities to actual outcomes. While traditional metrics such as accuracy and AUC focus on a model’s discriminatory power, the Brier score provides valuable insight into the reliability of the predicted probability. A lower Brier score signifies better calibration, indicating that the predicted probabilities closely match the true outcomes. This distinction is especially important in medical contexts, where accurate probability estimates can significantly impact decision-making and patient care.

### 2.5. Machine Learning Models

In this study, we evaluated eleven machine learning (ML) algorithms to predict early-stage risk of cardiogenic shock, treated as a binary outcome (1 = at risk, 0 = not at risk). These models were selected for their complementary strengths in analyzing complex medical data.

The ML framework was implemented in Python (The Python Software Foundation, Beaverton, OR, USA) (v3.11.11) using Google Colab (Alphabet Inc., Mountain View, CA, USA; accessed on 5 March 2025). Key libraries included pandas, matplotlib, seaborn, and scikit-learn. Data from five clinical phases were split into training (80%) and testing (20%) sets using train_test_split. Each model was configured with relevant hyperparameters for consistency. For example, LR was set with max_iter = 1000; ensemble models (RF, ET, GBC, ADA) used n_estimators = 100 and random_state = 42; SVM used an RBF kernel with C = 1.0; KNN had n_neighbors = 5. StandardScaler was applied to normalize all input data before model training.

Model performance was evaluated using key metrics: accuracy, precision, recall, F1-score, and Matthews correlation coefficient. Confusion matrices were also generated to better understand classification outcomes.

### 2.6. The Framework

**Figure 5:**
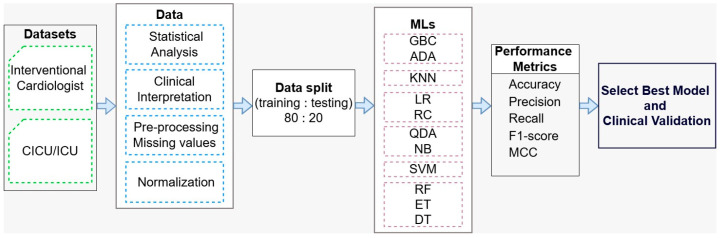
Machine learning-based framework for cardiogenic shock prediction.

The framework for cardiogenic shock detection based on machine learning is illustrated in Figure 5, highlighting key stages in the predictive modeling process.

Two datasets (marked with a green color) with real clinical data were used: interventional cardiologist and CICU units. These datasets capture the patient’s progression through various stages of care. The data were systematically processed through a series of steps, including statistical analysis, clinical interpretation, handling of missing values, and normalization (each marked in blue), to ensure consistency and quality throughout the study. The pre-processed data were split into training and testing sets in a 80:20 ratio for training and testing of eleven ML models (marked with a purple color) such as: tree-based (Extra Trees—ET, Random Forest—RF, Decision Tree—DT), probabilistic (Quadratic Discriminant Analysis—QDA, Naïve Bayes—NB), margin-based (Support Vector Machine—SVM), linear models (logistic regression—LR, Ridge Classifier—RC), distance-based (K-Nearest Neighbors—KNN), and boosting methods (Gradient Boosting—GBC, AdaBoost—ADA). To ensure reliable model selection and reduce the risk of overfitting, 5-fold cross-validation was applied exclusively to the training data. The training set was divided into five subsets: in each fold, four subsets were used for training, while the remaining subset was used for validation. Hyperparameters were optimized based on the average performance across the validation folds. Once the model was finalized through cross-validation, it was retrained on the full training set and evaluated on the hold-out 20% test set to measure its ability to generalize to new data.

These models are evaluated using standard performance metrics (accuracy—ACC, precision, recall, F1-score, and Matthews correlation coefficient) to identify the most ac-curate and clinically relevant model. The final step involved selecting the best-performing model, which was then subjected to clinical validation to ensure its reliability and applicability in real-world clinical settings.

To enhance the transparency and clinical utility of the selected machine learning models, we further integrated Explainable Artificial Intelligence (XAI) techniques into the evaluation process. While traditional metrics such as accuracy and F1-score quantify overall model performance, they do not provide insight into the reasoning behind specific predictions. In a critical care context—such as predicting cardiogenic shock—clinicians require more than just output labels; they need to understand the underlying decision logic. For this reason, we employed LIME (Local Interpretable Model-Agnostic Explanations) to generate individualized explanations of model predictions. LIME enables us to identify which clinical features most strongly influenced each prediction, distinguishing between factors that pushed the model toward a positive (high-risk) or negative (low-risk) outcome. This interpretability is especially valuable in clinical decision-making, as it highlights actionable variables and validates the relevance of model outputs in real-world scenarios. Ultimately, the combination of robust predictive performance and interpretability supports the deployment of machine learning tools in high-stakes environments like intensive and interventional cardiology units.

## 3. Results

### 3.1. Interventional Cardiologist Phase

In the interventional cardiologist phase, which begins after the patient has been evaluated in the emergency department and referred for coronary angiography or stent placement, the aim is to support interventional cardiologists in identifying patients at high risk of developing cardiogenic shock by analyzing clinical and laboratory parameters available at this stage. The model incorporates a comprehensive set of features, the parameters were described in the Methods section. These variables were selected to reflect both the hemodynamic and ischemic burden of the patient and will be processed using machine learning algorithms to identify the most relevant predictors of cardiogenic shock at this stage.

The analysis focuses on the most important coefficients determined by Random Forest, which are crucial for predicting the risk of cardiogenic shock in this phase. These include Killip class, reperfusion type, number of DES, potassium, lesion type (culprit lesion), CKI, and TIMI flow before PCI. These factors significantly influence the predictive power of the model, emphasizing the need to consider them in clinical decision-making.

In the context of the interventional cardiologist phase, the Random Forest (RF) model demonstrates strong predictive performance. With an accuracy of 87.10% (95% CI: 75.30–98.90), high sensitivity (85.71%), and specificity (88.24%), along with an AUC of 0.9496, the model effectively distinguished patients at risk. Notably, it also achieved a low Brier score of 0.1110, indicating well-calibrated probability estimates essential for guiding therapeutic decisions in high-stakes settings such as the cardiac catheterization lab. In comparison, the logistic regression model achieved a lower accuracy of 74.19% (95% CI: 58.79–89.60%) and a higher Brier score of 0.1567, suggesting that its predictions were less reliably calibrated. Although both models performed well in terms of classification metrics, Random Forest demonstrated superior calibration and overall predictive reliability, as shown in Table 1.

Additionally, the McNemar test *p*-value of 0.6171 indicates no statistically significant difference between the performance of the two models. This suggests that while RF outperforms LR in sensitivity, specificity, and F1-score, the difference in overall model performance is not large enough to be considered statistically significant, and both models can be used interchangeably in clinical practice depending on the specific needs for predictive power versus model interpretability.

Key parameters, including clinical factors such as Killip class, reperfusion type, number of DES, potassium, culprit lesion, CKI, and TIMI flow before PCI, were evaluated through logistic regression to assess their contribution to predicting the risk of cardiogenic shock in patients undergoing interventional cardiology procedures.

The results from the logistic regression analysis, as illustrated in Table 2, provide significant insights into the prediction of cardiogenic shock risk. Killip class (Coef = 1.1524, *p*-value = 0.0000) emerges as a highly significant predictor, with a positive coefficient indicating that a higher Killip class, reflecting a worse clinical condition, substantially increases the likelihood of poor outcomes such as cardiogenic shock. Similarly, reperfusion type (Coef = 1.3812, *p*-value = 0.0029) is significantly associated with improved patient outcomes, where successful reperfusion (e.g., through PCI) significantly reduces the risk of complications and enhances recovery. On the other hand, number of DES (Coef = 0.0795, *p*-value = 0.5944) does not show statistical significance, suggesting that the number of DES placed may not play a critical role in determining patient outcomes in this context. Potassium (K) (Coef = 0.4891, *p*-value = 0.2147) exhibits a positive association with outcomes, but this relationship is not statistically significant, indicating that potassium levels may not be a primary predictor in this scenario. Culprit lesion (Coef = 0.1261, *p*-value = 0.1045) also fails to reach statistical significance, although its inclusion provides valuable information regarding the severity of occlusion. Notably, Creatine Kinase Index (CKI) (Coef = −0.0006, *p*-value = 0.0052) is identified as a statistically significant negative predictor, with lower CKI levels correlating with poorer outcomes, which aligns with its established role in myocardial injury. Finally, TIMI flow before PCI (Coef = 0.0230, *p*-value = 0.9337) does not significantly predict patient outcomes in this phase, suggesting that the level of myocardial ischemia prior to intervention may not be a key factor in determining post-procedure prognosis. These findings highlight the critical role of clinical parameters such as Killip class and reperfusion type in predicting outcomes, while emphasizing the limited predictive value of other factors like number of DES and TIMI flow before PCI in the context of cardiogenic shock prediction.

To assess multicollinearity in the model and ensure the stability of the regression coefficients, we performed a Variance Inflation Factor (VIF) analysis for each of the key variables included in the model. The VIF values provide insight into how much the variance of a regression coefficient is inflated due to collinearity with other variables. A VIF value greater than 10 would typically suggest high multicollinearity, potentially leading to unreliable coefficient estimates. The following table summarizes the VIF values for each parameter, allowing us to assess the degree of multicollinearity and its potential impact on the model’s stability.

The Variance Inflation Factor analysis, as shown in Table 3, indicates that multicollinearity is not a significant concern in the model, as all VIF values are below the commonly accepted threshold of 10. However, higher VIF values for “TIMI flow before PCI”. (4.7122) and “culprit lesion” (4.4707) suggest some degree of overlap in the information they provide regarding the severity of coronary artery disease. Despite this, these variables continue to independently contribute to the predictive model, emphasizing their relevance in assessing the risk of cardiogenic shock.

Overall, this study’s findings, with their significant implications for medical decision-making, underscore the clinical utility of combining clinical and angiographic data in predicting cardiogenic shock and other adverse outcomes during the interventional cardiology phase. In this context, it is essential to recognize the importance of considering all seven key parameters—Killip class, reperfusion type, number of DES, potassium (K), culprit lesion, CKI, and TIMI flow before PCI. While three parameters may appear most representative based on their individual coefficients and statistical significance, disregarding any of the other critical parameters could lead to incomplete risk stratification and suboptimal patient management. Each parameter contributes a unique perspective to the model, reflecting different facets of the patient’s condition. For example, while Killip class and reperfusion type provide valuable insights into the severity of heart failure and the effectiveness of treatment, the potassium level and TIMI flow before PCI score offer additional biochemical and pre-treatment insights that are equally essential in predicting patient outcomes. Additionally, the culprit lesion and CKI, although statistically less significant on their own, still play a role in providing a fuller understanding of myocardial injury and recovery potential.

Thus, the integration of these seven parameters in the predictive model ensures that clinicians can more accurately assess the risk of cardiogenic shock and other adverse events.

To support the conclusion that it is important to consider all seven parameters, we can analyze the predictive performance of the Random Forest (RF) model using both all seven features and only the top three features.

When using all seven features, the model demonstrates a higher overall accuracy of 87.10%, with strong sensitivity (88.24%) and specificity (85.71%), as well as an AUC of 94.96%. These results indicate that the model is well-calibrated and can effectively predict the risk of cardiogenic shock while minimizing both false positives and false negatives. The F1-score of 86.96% further reflects the model’s ability to balance precision and recall, essential for predicting high-risk patients.

In contrast, when the model is limited to just the top three features, accuracy decreases to 80.65%, and the F1-score drops to 80.00%, demonstrating a reduction in the model’s overall predictive capability. Although the sensitivity increases to 92.31%, which indicates better detection of high-risk patients, specificity drops to 72.22%, meaning there are more false positives, and the model is less reliable in identifying low-risk patients. The AUC also drops to 0.8172, further supporting the notion that using fewer features compromises the model’s ability to discriminate effectively between high-risk and low-risk patients.

These findings emphasize the importance of using all seven parameters in the predictive model, as omitting any of them can lead to a decrease in model performance, particularly in terms of accuracy and overall discrimination. The comprehensive inclusion of all features ensures a more robust and reliable model, which is critical in clinical decision-making, especially for managing high-risk patients in the interventional cardiology phase. Therefore, even though some parameters may appear less significant individually, their collective contribution is indispensable for achieving optimal model performance and improving clinical outcomes.

### 3.2. Cardiac Intensive Care Unit

The prediction of cardiogenic shock in patients within the cardiac intensive care unit is a critical element of personalized medical care. Early identification of at-risk patients allows for prompt intervention, which can significantly improve clinical outcomes.

The initial step in our analysis involves considering a comprehensive set of 45 key parameters in assessing patients during the CICU, covering demographic data, cardiovascular risk factors, clinical and biological parameters, ECG findings, echocardiographic findings, and angiographic parameters, with additional data such as the occurrence of cardiac arrest during hospitalization, events of resuscitation during hospitalization, ICU admission, the need for ventilatory support, and the use of inotropic and vasopressor support. Each of these factors plays a significant role in predicting patient outcomes and informing clinical decisions in this critical phase.

Using Random Forest, we perform feature selection to identify the most representative key parameters that are most predictive of cardiogenic shock. These key parameters, which include Killip class, hemoglobin, pain onset, heart rate, age, urea, and sex, are then used in the predictive models. The subsequent prediction is performed using two modeling approaches, logistic regression and Random Forest, to assess the effectiveness of the selected key parameters in predicting cardiogenic shock outcomes, as demonstrated in Table 4.

The Random Forest model outperforms the logistic regression model across all performance metrics, including accuracy, sensitivity, F1-score, and AUC. With an accuracy of 80.77%, Random Forest demonstrates a higher ability to correctly classify both positive and negative outcomes compared to logistic regression, which has an accuracy of 76.92%. The sensitivity of Random Forest (80.00%) is also higher than that of logistic regression (73.33%), meaning it is better at identifying patients at high risk of cardiogenic shock.

Specificity remains the same for both models at 81.82%, indicating that both models are equally effective in identifying patients at low risk. However, the higher F1-score of Random Forest (0.8090) compared to logistic regression (0.7734) demonstrates that Random Forest provides a better balance between precision and recall.

Finally, the AUC of Random Forest (0.8667) is superior to that of logistic regression (0.7818), suggesting that Random Forest has a better overall discriminatory ability in predicting cardiogenic shock outcomes.

The *p*-value of 1.0000 from McNemar’s test indicates that there is no statistically significant difference in misclassification between the two models. This suggests that while the Random Forest model has a slightly higher AUC, both models have comparable performances, and the choice of model may depend on other factors such as interpretability and computational efficiency.

The analysis of logistic regression coefficients reveals that Killip class is a highly significant predictor for the risk of cardiogenic shock, as demonstrated by its low *p*-value and positive coefficient. Although other variables such as hemoglobin, pain onset, heart rate, age, and urea are included in the model, none of them show significant associations with the outcome, as evidenced by their high *p*-values, as shown in Table 5.

However, these variables may still contribute to the predictive model, as their VIF indicate low to moderate levels of multicollinearity, suggesting that they do not significantly overlap with other predictors. For instance, hemoglobin and BUN have reasonable VIFs, but their lack of statistical significance (with *p*-values above 0.05) suggests that they do not strongly influence the prediction of cardiogenic shock in this model.

Thus, while Killip class remains the most significant parameter for predicting cardiogenic shock, the potential importance of the other variables in specific clinical contexts should not be overlooked, as shown in Table 6. These factors, despite not being significant in this analysis, may still hold predictive value when considered in combination with other clinical parameters or in more refined models.

Given the inclusion of numerous variables (45 parameters), Random Forest (RF) was utilized for feature selection, focusing on the top seven most important predictors. The model performance using all seven features achieved an accuracy of 0.8077, with a 95% CI for accuracy ranging from 0.6154 to 0.8846. In contrast, when the model was restricted to the top one parameter (Killip class), the performance dropped significantly, with an accuracy of 0.7692, sensitivity of 0.6923, and AUC of 0.7758. These results highlight that, while a single highly significant predictor can offer some predictive value, using all seven key parameters—such as Killip class, cardiac arrest, and mechanical complications—leads to a more comprehensive and reliable assessment of cardiogenic shock risk. The full model consistently outperforms the simplified version, demonstrating that each parameter provides distinct and valuable information. Therefore, incorporating all relevant parameters is essential for enhancing prediction accuracy and enabling timely, targeted interventions in the cardiac ICU.

In this study, Random Forest was used not only as a classifier but also for identifying the most predictive clinical parameters associated with cardiogenic shock in the cardiac intensive care unit. With a Brier score of 0.1505, the model demonstrated both high discriminative ability and reliable probability estimates. This level of calibration is particularly relevant in medical contexts, where accurate risk prediction is critical for guiding clinical decisions. In comparison, the logistic regression model, although showing competitive accuracy and AUC, had a higher Brier score of 0.1870, suggesting less precise probability estimates. These findings support the use of calibrated models like Random Forest for clinical decision support where both classification accuracy and risk reliability are essential.

The Random Forest model, enhanced through feature selection and VIF analysis, offers a strong balance between predictive performance and interpretability. Identifying key variables not only improves model stability but also contributes to building practical tools that support early recognition of patients at risk of cardiogenic shock.

To further strengthen model reliability, it is important to explore additional algorithms. Models such as Extra Trees and Decision Trees may better capture feature interactions, while probabilistic approaches like QDA and Naïve Bayes can improve understanding of prediction uncertainty. Support Vector Machines, K-Nearest Neighbors, and ensemble methods like Gradient Boosting and AdaBoost offer complementary strengths that may enhance predictive accuracy and generalizability.

Overall, by combining key feature selection with diverse machine learning strategies, this approach supports the development of more accurate and reliable predictive tools for early detection of cardiogenic shock. These tools can improve clinical outcomes by enabling timely and targeted interventions.

Table 7 presents the performance results for the interventional cardiologist dataset (BD_ES_Interventional.csv). Among the 11 evaluated ML models, RF and QDA achieved the highest accuracy, both reaching 87.50%. These two models also have identical values for precision, recall, and F1-score, each of 87.50%. In terms of MCC, RF slightly out-performed QDA, with RF achieving 75.59% and QDA obtaining 75.00%. In comparison, NB have the lowest performances: ACC: 71.87%, Precision: 74.24%, Recall: 71.87%, F1-score: 71.17%, MCC: 46.05%.

Table 8 shows the results for the CICU dataset (BD_ES_CICU.csv). From all MLs, RF and QDA demonstrate the best performance. For RF, the metrics are: ACC: 84.37%, precision: 85.62%, recall: 84.37%, F1-score: 84.23%, and MCC: 69.99%. Similarly, QDA achieves ACC: 84.37%, precision: 84.50%, recall: 84.37%, F1-score: 84.35%, and MCC: 68.88%. On the other hand, ADA shows the lowest performance, with ACC, precision, recall, and F1-score all at 68.75%, and an MCC of 37.50%.

As observed QDA was the only model that demonstrated consistently strong performance across all evaluation metrics.

Notably, QDA was the only model that consistently demonstrated strong performance across both interventional and intensive care phases, suggesting its robustness and potential for generalized application in dynamic STEMI-CS risk stratification. Our findings support the use of RF and QDA in advanced care phases, offering timely risk estimation that may guide decisions such as extended CICU monitoring, early mechanical support, or stepped-down care in lower-risk patients.

Moreover, the XAI-LIME interpretation of the Random Forest classifier during the interventional cardiologist phase provides a detailed view of how individual features influenced the model’s prediction. The tables below, Table 9 and Table 10, integrates global and local interpretability insights from the Random Forest model used to predict cardiogenic shock. It summarizes both the most influential features identified across the entire dataset (global) and the instance-specific contributions revealed by XAI-LIME (local), including directionality and clinical significance.

## 4. Discussion

This study evaluated predictive models for cardiogenic shock progression in STEMI patients during the post-revascularization period, focusing on the interventional cardiology and CICU phases. Using machine learning algorithms—particularly RF and QDA—we aimed to enhance risk stratification during these advanced stages. RF outperformed logistic regression, while QDA demonstrated consistent performance across both phases, highlighting its potential for generalizable, phase-independent application.

Model selection was adapted to the clinical characteristics of each phase. In the catheterization lab, RF integrated key angiographic variables. In the CICU, its accuracy remained high by incorporating complex hemodynamic and laboratory data. Although not always the top performer in individual stages, QDA’s stable results across all five clinical phases support its utility in dynamic risk prediction.

Unlike most studies based on logistic regression, our approach emphasizes the predictive value of AI-based models in identifying patients at risk before shock occurs. This shift from reactive to proactive risk assessment enables earlier, targeted interventions for vulnerable STEMI patients.

In the post-revascularization phase, predictive tools are particularly valuable for optimizing CICU resources. Even after reperfusion, patients remain at risk due to hemodynamic instability. Predictive models can support clinical decisions on monitoring intensity and guide safe transfers to general wards, improving both outcomes and resource use. As a key outcome, this study introduced the P-E-I-CI model—a novel, author-designed framework for continuous, phase-adapted risk stratification in STEMI-CS.

### 4.1. Comparison with Existing Risk Models

Bai et al. developed a nomogram based on machine learning algorithms—including LASSO, SVM, LightGBM, and XGBoost—to predict in-hospital cardiogenic shock in STEMI patients, showing superior performance compared to traditional clinical scores [42]. While their model focused on overall hospitalization, our study brings a novel contribution by targeting the interventional cardiology and CICU phases. Using interpretable models like Random Forest and QDA, we enable dynamic, phase-specific risk reassessment after revascularization, directly supporting decisions related to care escalation and ICU resource allocation. Similarly, Böhm et al. introduced the STOP SHOCK score, a validated clinical tool incorporating 13 routinely available variables—including vital signs, lab markers, oxygen saturation, and mechanical ventilation requirements—to assess shock risk early after hospital admission [9]. Their model aims to assist triage at presentation or immediately post-revascularization. Our approach complements this by addressing the critical post-angioplasty period, using machine learning to build the phase-adapted P-E-I-CI framework for continuous risk monitoring during later stages of STEMI care.

Lim et al. proposed the R-O-S-E model, which outlines four hemodynamic management phases in cardiogenic shock: Recognize/Rescue, Optimization, Stabilization, and De-escalation/Exit Therapy [5]. Our work aligns with the early recognition and rescue stages, consistent with the SCAI SHOCK classification, and advances the concept by developing phase-specific predictive tools. By identifying key variables associated with CS progression, we aim to enable earlier risk stratification and more timely therapeutic interventions, potentially preventing further clinical deterioration.

### 4.2. Dynamic Risk Assessment vs. Classical Clinical Scores

Accurate and early identification of cardiogenic shock is essential for improving outcomes. The SCAI SHOCK classification provides a five-stage framework, starting with Stage A (at-risk patients, such as those with STEMI) and progressing to Stage E (refractory shock or death) [4]. Stage B, representing early shock, is especially critical for timely recognition of hemodynamic deterioration. Our study aligns with this phased approach by aiming to intervene early—ideally during Stage B—while also extending risk monitoring beyond the acute phase, into the post-revascularization period. This extension is clinically important, as reassurance following angioplasty may mask ongoing risk during the interventional cardiology and CICU phases.

Unlike complex ICU scoring systems such as APACHE or SOFA—which use 10 to 20 variables for mortality prediction [43,44,45]—the P-E-I-CI framework is not a numerical score but a dynamic evaluation method based on stage-specific predictive models. Each model uses a limited set of three to five routinely collected variables. While our initial analysis explored more complex models with numerous inputs, we found that simpler configurations achieved better generalizability and clinical usability. This approach offers several practical advantages: improved feasibility for real-time clinical use, reduced data collection burden, faster risk estimation, and adaptability across various healthcare settings. Importantly, P-E-I-CI promotes continuity in risk assessment, allowing clinicians to monitor patients consistently across phases without switching tools or recalibrating scores. This makes the model not only clinically relevant, but also scalable and implementable in both high-resource and resource-limited environments.

### 4.3. Phase-Tailored Models for Personalized STEMI-CS Management

This study emphasizes the need for machine learning models tailored to each clinical phase in managing STEMI complicated by cardiogenic shock. Model performance varied by stage, with Random Forest and QDA showing strong results in interventional cardiology and intensive care phases by effectively integrating angiographic and ICU-specific hemodynamic parameters.

Key predictors—such as Killip class, biochemical markers, and angiographic indicators—proved valuable for risk stratification and guiding personalized interventions. By analyzing each phase separately, the study highlights the importance of context-sensitive prognostic models adapted to the specific clinical setting throughout the continuum of care.

### 4.4. Interventional Cardiologist Phase

Using the Random Forest (RF) algorithm, we developed a predictive model for the angiography stage, where the on-call interventional cardiologist can continue the dynamic evaluation of STEMI patients and estimate the risk of progression to cardiogenic shock based on relevant variables.

Killip class remains the most important predictor, which can be explained by the fact that patients in Killip classes 3 and 4 often cannot tolerate the supine position, an essential posture for performing angiography. The next most significant parameter is the type of reperfusion, which has a major impact on patient outcomes. Patients who received initial fibrinolysis may have a partially reopened infarct-related artery, whereas those undergoing primary PCI typically present with a total occlusion [19]. These two parameters are followed by the number of DES implanted, as a longer segment of occlusion requires more stents, which in turn implies a longer procedure duration, prolonged ischemia time, and increased exposure to contrast agent [46,47]. The fourth parameter is potassium level, which shows a similar level of importance to the location of the occlusion in the epicardial coronary arteries, CKI, and TIMI flow before PCI.

The results of our study, highlighting Killip class as the most powerful predictive variable during angiography, are also supported by the literature. Wei Z. et al. published a study involving 625 patients, later validated on an additional cohort of 245 patients, demonstrating that both Killip class and shock index are independent predictors of cardiogenic shock. Furthermore, the shock index showed high accuracy in identifying the risk of CS during PCI [48].

Regarding the applicability of the predictive model, factors such as the location of the occlusion [49,50,51], the number of DES implanted [52], and TIMI flow before PCI [53,54,55] flow are well-known variables that can influence the risk of progression to cardiogenic shock, depending on their severity [42].

Thus, Killip class is confirmed as the clinical variable with the highest predictive power at this stage, highlighting its importance in the evaluation and management of STEMI patients during PCI. This finding supports its integration into clinical risk stratification algorithms, contributing to the optimization of therapeutic decisions and the prevention of severe complications.

In conclusion, the predictive model developed for the interventional cardiology phase highlights the continued utility of early clinical and procedural variables—particularly Killip class and reperfusion strategy—in stratifying patients at risk for cardiogenic shock. These findings support the role of ML-driven tools in guiding therapeutic decisions during PCI and improving intra-procedural risk management.

### 4.5. Cardiac Intensive Care Unit/Intensive Care Unit Phase

In the post-revascularization phase, patients require close monitoring in the CICU or even ICU, which is why we considered it appropriate to develop a predictive model specifically for this stage. This is the phase during which patients may either be monitored for progression to cardiogenic shock or may already be experiencing it and receiving targeted treatment.

In the RF model, Killip class remained the most important predictive variable, supporting the conclusions of previous studies that highlight the severity of heart failure as the primary determinant of CS risk.

Peters E.J. and colleagues developed and externally validated a clinically interpretable risk score aimed at predicting in-hospital mortality among patients with cardiogenic shock admitted to cardiac intensive care units. Their model incorporated routinely available parameters such as blood urea nitrogen, minimum oxygen saturation, lowest systolic blood pressure, mechanical ventilation requirement, age, and maximum anion gap, demonstrating superior performance compared to traditional scoring systems [56]. While their work provides a valuable tool for outcome stratification and mortality risk communication, our study focuses instead on the early prediction of cardiogenic shock as a clinical complication in STEMI patients, particularly during the interventional and CICU phases. By identifying patients at risk before hemodynamic collapse occurs, our machine learning–based approach supports proactive clinical decision-making and escalation of care, with the ultimate goal of preventing deterioration and improving survival outcomes, rather than estimating mortality once cardiogenic shock has already developed.

Shen et al. explored the use of machine learning to phenotype cardiogenic shock, identifying distinct clinical subgroups with specific characteristics and prognostic outcomes in the post-revascularization setting. While their work highlights the value of unsupervised learning for retrospective classification and treatment personalization after shock onset, our study brings a novel contribution by focusing on early prediction, before CS occurs [57]. Specifically, we developed supervised, phase-specific models targeting the interventional and CICU phases, enabling real-time identification of patients at risk for hemodynamic deterioration after PCI. This proactive approach shifts the focus from classification after diagnosis to anticipation and prevention, with the goal of supporting timely therapeutic escalation and improving clinical outcomes in high-risk STEMI patients.

Killip class proved to be the most powerful predictor of progression to CS, maintaining its relevance across all analyzed stages, across both the interventional cardiology and CICU phases. In post-revascularization period, the predictive model allows for careful monitoring of revascularized patients, anticipating potential complications.

Following Killip class, the second most important parameter in the predictive model for the CICU stage was serum hemoglobin level. A pathophysiological explanation for this finding is that in anemic patients, the reduction in hemoglobin and oxygen-carrying capacity leads to decreased oxygen delivery to cardiomyocytes, favoring myocardial ischemia. This hypoxia triggers a compensatory response through activation of the sympathetic nervous system and the renin–angiotensin–aldosterone system, leading to increased heart rate and blood volume, which overload the heart and worsen ischemia. Additionally, the heightened inflammation associated with anemia may contribute to plaque destabilization and the formation of coronary thrombus.

Bai Z. et al. demonstrated that hemoglobin is a predictive marker of progression to CS in STEMI patients; however, their study focused on the very early phase, starting from admission and prior to revascularization [42]. This result was expected, as anemia worsens myocardial ischemia. Unlike the study by Bai Z., our study supports the use of hemoglobin as a predictive marker for STEMI-CS specifically in the post-revascularization phase. Thus, the conclusions of the two studies suggest that hemoglobin could serve as a predictive marker over a broader time frame, both in the acute phase and during post-revascularization monitoring. Bai Z. et al. demonstrated the predictive value of hemoglobin using the LASSO model, where Hb was found to be significant alongside age, aspartate aminotransferase (AST), lactate dehydrogenase (LDH), white blood cell count (WBC), chronic kidney disease (CKD), and delayed hospital presentation [42]. In our model, developed using Random Forest (RF), hemoglobin was included alongside time from pain onset, heart rate, age, urea, and sex.

Regarding the variable “time from pain onset” (or delayed hospital presentation, as referred to by Bai Z.), the results of our study are consistent with those of Bai Z., who identified delayed hospital arrival as an important predictive marker for STEMI-CS, primarily due to prolonged ischemia time and increased necrotic area. In his study, Bai Z. examines two key aspects: patient delay and in-hospital delay. While in-hospital delays can be reduced through the development of specialized STEMI centers, patient delays can only be addressed by increasing public awareness. In this regard, public education campaigns would be highly beneficial, enabling individuals to recognize the symptoms of a heart attack and seek medical attention as quickly as possible.

The variable “time from pain onset” was also analyzed in a large-scale study involving 6838 patients, conducted by Auffret V. et al., who validated a score called the ORBI Score using logistic regression (LR). The results demonstrated that delayed hospital presentation is a strong predictor for STEMI-CS [58]. The conclusions of that study, combined with the findings from our own research—confirming the relevance of this parameter both in the post-revascularization phase and in the acute stage of STEMI—suggest that time from pain onset is a relevant predictor for both early and late progression to cardiogenic shock.

Regarding the contribution of the “heart rate” parameter in the CICU predictive model, our results align with the existing literature. Bohm A. et al., in a study published in January 2025, used LR to create ACS-CS predictive models, demonstrating that heart rate is a significant predictor. However, their model included a different set of parameters compared to ours, specifically hypercholesterolemia, congestive heart failure, heart rhythm, shock index, type of ACS, respiratory rate, oxygen saturation, blood glucose, systolic blood pressure, age, history of hypertension, and sex [9].

Another retrospective study conducted by Jentzer et al. on a cohort of 10,000 patients demonstrated that heart rate is a risk predictor for ACS-CS. Additionally, among ACS patients in the pre-shock phase, several factors were identified as being associated with a higher risk of death, including hypotension, elevated blood glucose, and a high shock index [59].

Both studies support the use of heart rate as a predictor for AMI-CS; however, our study reinforces the value of this parameter specifically in the STEMI-CS subgroup, demonstrating its importance even in the post-revascularization phase.

Studies that analyze “age” as a predictive factor for STEMI-CS progression are limited, with most focusing on patients with AMI-CS in general. Nevertheless, Chang Y. et al. published a study in which, using both LR and LASSO, they demonstrated that age—alongside AST, CKD, hemoglobin, WBC, and shock index—is a significant predictor of cardiogenic shock in STEMI patients [60].

Thus, the results of our study support the idea that surveillance in the CICU should be based not only on established clinical parameters but also on additional predictors such as hemoglobin, time from pain onset, and heart rate. These factors can enhance risk stratification and guide the management of STEMI patients.

In summary, the CICU model confirms the prognostic value of clinical variables such as hemoglobin, time from symptom onset, and heart rate in the post-revascularization setting. These results reinforce the need for continued risk monitoring in intensive care and support the integration of ML-based tools for optimizing surveillance, resource allocation, and targeted interventions in high-risk STEMI patients.

In addition to the predictors analyzed in our CICU model, recent evidence highlights the prognostic importance of in-hospital bleeding (IHB) in ACS patients. Spadafora et al. (2025) demonstrated that IHB is independently associated with increased one-year mortality, recurrent infarction, and major bleeding events, marking it as a significant indicator of clinical frailty [61]. Although not included in our current models, incorporating IHB into future predictive algorithms may improve their accuracy and real-world applicability, particularly in the post-revascularization phase where patients remain vulnerable to both ischemic and hemorrhagic complications.

## 5. Limitations

Several limitations of this study should be considered when interpreting its findings:

- Sample Size and Generalizability—As a single-center study conducted in Romania, the relatively modest number of included patients may affect the generalizability of the results. Future validation across larger, multicenter cohorts is necessary to confirm model performance in diverse clinical settings.
- Ethnic and Geographic Homogeneity—All patients included in the cohort were Caucasian and treated in an Eastern European healthcare environment. This limits the extrapolation of the findings to populations of different ethnic backgrounds or to health systems with broader access to advanced therapies.
- Resource Availability—The study was carried out in a university-affiliated tertiary center with relatively high resource availability. Predictive model performance might differ in smaller regional hospitals with limited access to cardiac intensive care or catheterization laboratories.
- Need for External Validation—The proposed models, including the P-E-I-CI framework, were developed and evaluated on a single dataset. Prospective studies and external validation on independent cohorts are essential to establish their clinical utility and robustness

Despite these limitations, this study contributes to the growing body of evidence supporting dynamic, phase-specific risk assessment in STEMI-CS. The findings offer a foundation for further refinement and prospective testing in multicenter, international cohorts, which would allow a better understanding of how patient outcomes may be influenced by systemic, regional, or socioeconomic differences.

## 6. Future Research Priorities

The predictive models discussed in this study require external validation and could serve as a starting point for future multicenter research on independent patient cohorts from diverse healthcare systems and regions worldwide. This would provide a more comprehensive understanding of how social and geographical factors influence the progression of STEMI-CS.

Looking ahead, an essential focus of further research should be the development of predictive models for life-threatening tachyarrhythmias in STEMI patients. Alongside arrhythmias, IHB represents another critical complication with significant prognostic impact, as recent studies have shown. Including IHB as a target variable in future models may enhance their clinical relevance, especially for patients in the post-revascularization phase who remain at high risk for both ischemic and hemorrhagic events. Addressing these challenges could significantly impact patient outcomes by enabling earlier intervention in high-risk cases.

Moreover, the use of predictive modeling to guide personalized treatment approaches aligns with current trends in precision medicine. As this field evolves, adapting these models to individual patient profiles may lead to more effective and tailored therapeutic strategies.

Despite the promising potential of the current models, there is a clear need for further research to incorporate advanced deep learning techniques, such as DeepSurv and Long Short-Term Memory (LSTM) models, into clinical predictive analytics. DeepSurv, designed specifically for survival analysis, could enhance predictive accuracy by capturing complex, nonlinear relationships between patient characteristics and survival outcomes, especially in cases involving censored data. Similarly, LSTM models, which are well-suited for processing time-series data and long-term dependencies, could offer deeper insights into the evolving progression of conditions like STEMI-CS. These models have the capacity to uncover patterns and trends that traditional machine learning approaches may overlook, thereby improving the precision of predictions and enabling more timely, personalized interventions.

These research directions converge toward a more adaptive clinical paradigm, grounded in prediction, personalization, and early intervention in STEMI-CS.

## 7. Translational Opportunities for Artificial Intelligence

The predictive models developed in this study open several promising directions for integrating artificial intelligence into real-world clinical workflows:

- Development of an easy-to-use clinical risk calculator for rapid assessment of cardiogenic shock risk in ambulance or emergency department settings.
- Creation of a mobile application that provides decision support based on the proposed predictive models, particularly useful in prehospital and early hospital phases.
- Integration of these models into hospital information systems to automatically alert interventional teams (e.g., ECMO, intra-aortic balloon pump, intensive care support).

Implementing AI-based decision support tools in clinical settings could enable earlier and more precise interventions, reduce practice variability, and ultimately improve outcomes for STEMI patients at risk of cardiogenic shock.

## 8. Conclusions

This study proposes a comprehensive and dynamic approach to predicting the risk of progression to cardiogenic shock in STEMI patients, integrating commonly available clinical and paraclinical parameters across distinct phases of care. Unlike static risk scores, the P-E-I-CI model enables continuous, phase-specific risk stratification, from initial medical contact to the catheterization laboratory and cardiac intensive care unit. By prioritizing early detection and targeted intervention, the model has the potential to enhance clinical decision-making and improve patient outcomes.

Clinically, the model supports personalized post-revascularization monitoring using simple yet powerful predictors such as Killip class, hemoglobin, time from symptom onset, and heart rate. Its implementation into clinical workflows could reduce diagnostic delays, optimize resource allocation, and decrease mortality and complications related to cardiogenic shock.

The integration advanced deep learning techniques, such as DeepSurv and LSTM models in future research could significantly advance our ability to predict patient outcomes and enhance clinical decision-making in dynamic, time-sensitive medical contexts. This work represents a meaningful advancement toward proactive, patient-centered STEMI management, with the ultimate goal of improving both immediate and long-term outcomes in high-risk populations.

## Figures and Tables

**Table 1:** Model performance comparison of interventional cardiologist phase.

Model	Accuracy	Sensitivity	Specificity	F1-Score	AUC	Brier Score
Random Forest	0.8710	0.8571	0.8824	0.8696	0.9496	0.1110
Logistic Regression	0.7419	0.7857	0.7059	0.7437	0.8908	0.1567

**Table 2:** Coefficients (logistic regression).

Parameter	Coefficient	*p*-Value	95% Confidence Interval
Killip class	1.1524	0.0000	[0.5899, 1.7148]
Reperfusion type	1.3812	0.0029	[0.4634, 2.2991]
Number of DES	0.0795	0.5944	[−0.2162, 0.3752]
Potassium	0.4891	0.2147	[−0.2917, 1.2699]
Culprit lesion	0.1261	0.1045	[−0.0278, 0.2800]
Creatine Kinase Index	−0.0006	0.0052	[−0.0010, −0.0002]
TIMI flow before PCI	0.0230	0.9337	[−0.5240, 0.5700]

**Table 3:** Variance Inflation Factor (VIF).

Parameter	VIF
Killip class	1.1085
Reperfusion type	2.2669
Number of DES	3.5535
Potassium	1.1345
Culprit lesion	4.4707
CKI	1.0195
TIMI flow before PCI	4.7122

**Table 4:** Model performance comparison of the cardiac intensive care unit phase.

Model	Accuracy	Sensitivity	Specificity	F1-Score	AUC	Brier Score
Random Forest	0.8077	0.8000	0.8182	0.8090	0.8667	0.1505
Logistic Regression	0.7692	0.7333	0.8182	0.7734	0.7818	0.1870

**Table 5:** Coefficients (logistic regression).

Parameter	Coefficient	*p*-Value	95% Confidence Interval
Killip class	1.0570	0.0000	[0.5680, 1.5460]
Hemoglobin	−0.0236	0.8399	[−0.2558, 0.2085]
Pain onset	0.2926	0.2556	[−0.2182, 0.8033]
HR at presentation	0.0033	0.6937	[−0.0134, 0.0200]
Age	0.0255	0.2682	[−0.0202, 0.0713]
Blood urea nitrogen (BUN)	−0.0095	0.1578	[−0.0228, 0.0038]
Sex	0.5778	0.2839	[−0.4923, 1.6479]

**Table 6:** Variance Inflation Factor (VIF).

Parameter	VIF
Killip class	1.1371
Hemoglobin	1.3250
Pain onset	1.2307
HR at presentation	1.1263
Age	1.5625
BUN	1.4209
Killip class at presentation	1.1371

**Table 7:** Performance evaluation of 11 MLs on interventional cardiologist phase.

MLs	ACC	Precision	Recall	F1-Score	MCC	TN	FP	FN	TP
ET	0.78125	0.782353	0.78125	0.781036	0.563602	13	3	4	12
RF	0.87500	0.880952	0.87500	0.874510	0.755929	15	1	3	13
DT	0.75000	0.753968	0.75000	0.749020	0.503953	11	5	3	13
QDA	0.87500	0.875000	0.87500	0.875000	0.750000	14	2	2	14
NB	0.71875	0.742424	0.71875	0.711712	0.460566	14	2	7	9
SVM	0.78125	0.791498	0.78125	0.779310	0.572656	14	2	5	11
LR	0.75000	0.750000	0.75000	0.750000	0.500000	12	4	4	12
RC	0.75000	0.750000	0.75000	0.750000	0.500000	12	4	4	12
GBC	0.78125	0.782353	0.78125	0.781036	0.563602	12	4	3	13
ADA	0.78125	0.782353	0.78125	0.781036	0.563602	13	3	4	12
KNN	0.78125	0.791498	0.78125	0.779310	0.572656	14	2	5	11

**Table 8:** Performance evaluation of 11 MLs on cardiac intensive care unit phase.

MLs	ACC	Precision	Recall	F1-Score	MCC	TN	FP	FN	TP
ET	0.68750	0.687500	0.68750	0.687500	0.375000	11	5	5	11
RF	0.84375	0.856275	0.84375	0.842365	0.699913	12	4	1	15
DT	0.68750	0.687500	0.68750	0.687500	0.375000	11	5	5	11
QDA	0.84375	0.845098	0.84375	0.843597	0.688847	14	2	3	13
NB	0.78125	0.791498	0.78125	0.779310	0.572656	14	2	5	11
SVM	0.81250	0.817460	0.81250	0.811765	0.629941	12	4	2	14
LR	0.71875	0.719608	0.71875	0.718475	0.438357	11	5	4	12
RC	0.78125	0.782353	0.78125	0.781036	0.563602	12	4	3	13
GBC	0.78125	0.782353	0.78125	0.781036	0.563602	12	4	3	13
ADA	0.68750	0.687500	0.68750	0.687500	0.375000	11	5	5	11
KNN	0.75000	0.753968	0.75000	0.749020	0.503953	13	3	5	11

**Table 9:** Summary of global vs. local feature interpretability (Random Forest model—cardiogenic shock prediction in interventional cardiologist phase).

Interpretability Method	Feature	Contribution	Direction of Influence	Clinical Implication
Global Feature Importance (Random Forest)	Killip class; reperfusion type; CKI; TIMI flow before PCI; potassium; culprit lesion type; number of DES	N/A	Not directional	High general importance for model accuracy across all patients
Local Interpretation (XAI-LIME)	Killip class	+0.12	Toward Class 1 (Positive)	Indicates acute heart failure severity
Local Interpretation (XAI-LIME)	Sodium > 0.61	+0.04	Toward Class 1 (Positive)	Hyponatremia, which is associated with hemodynamic instability, may indicate severe left ventricular dysfunction
Local Interpretation (XAI-LIME)	Heart rate > 0.44	+0.02	Toward Class 1 (Positive)	Tachycardia is an early marker of hemodynamic instability
Local Interpretation (XAI-LIME)	CKI	Minor Positive	Toward Class 1 (Positive)	Elevated CK levels indicate extensive myocardial necrosis and severe ventricular dysfunction
Local Interpretation (XAI-LIME)	Diabetes mellitus	Minor Positive	Toward Class 1 (Positive)	Comorbid diabetes may elevate risk
Local Interpretation (XAI-LIME)	Primary PCI = −0.54	−0.11	Toward Class 0 (Negative)	Primary PCI may reduce shock risk
Local Interpretation (XAI-LIME)	Reperfusion type = −1.18	−0.07	Toward Class 0 (Negative)	Timely reperfusion mitigates severity
Local Interpretation (XAI-LIME)	Culprit lesion type = −1.20	−0.06	Toward Class 0 (Negative)	Culprit lesion linked to prognosis
Local Interpretation (XAI-LIME)	Fibrinogen = −2.07	−0.04	Toward Class 0 (Negative)	Inflammatory marker with prognostic value
Local Interpretation (XAI-LIME)	Potassium = −3.74	−0.03	Toward Class 0 (Negative)	Electrolyte imbalance affects cardiac function
Local Interpretation (XAI-LIME)	Creatinine = −1.79	−0.02	Toward Class 0 (Negative)	Reflects kidney function and metabolic stress

**Table 10:** Summary of global vs. local feature interpretability (Random Forest model—cardiogenic shock prediction in cardiac intensive care unit phase).

Interpretability Method	Feature	Contribution	Direction of Influence	Clinical Implication
Global Feature Importance (Random Forest)	Killip class, hemoglobin, pain onset, heart rate, age, urea, sex	N/A	Not directional	High general importance for model accuracy across all patients
Local Interpretation (XAI-LIME)	Killip class	+0.97	Toward Class 1 (Positive)	Indicates severity of heart failure at presentation
Local Interpretation (XAI-LIME)	Mitral regurgitation	+0.98	Toward Class 1 (Positive)	Mitral regurgitation suggests hemodynamic compromise
Local Interpretation (XAI-LIME)	Sex	+1.28	Toward Class 1 (Positive)	Sex-based risk variation in cardiac outcomes
Local Interpretation (XAI-LIME)	Heart rate	+0.74	Toward Class 1 (Positive)	Tachycardia is an early marker of hemodynamic instability
Local Interpretation (XAI-LIME)	Pain onset	+0.33	Toward Class 1 (Positive)	Delayed pain onset may reflect atypical presentation
Local Interpretation (XAI-LIME)	QRS complex duration	−0.59	Toward Class 0 (Negative)	Prolonged QRS predicts in-hospital mortality, cardiogenic shock, and need for circulatory support
Local Interpretation (XAI-LIME)	Location of ST-segment elevation on ECG	−0.49	Toward Class 0 (Negative)	Location of ST-segment elevation as a prognostic marker
Local Interpretation (XAI-LIME)	Resuscitated out-of-hospital cardiac arrest (OHCA)	−0.51	Toward Class 0 (Negative)	Resuscitated OHCA indicates severe ischemia and early ventricular dysfunction
Local Interpretation (XAI-LIME)	EKG rhythm	−0.45	Toward Class 0 (Negative)	ECG rhythm indicates electrical stability or severe myocardial damage
Local Interpretation (XAI-LIME)	ST elevation in aVR	−0.27	Toward Class 0 (Negative)	ST elevation in aVR linked to global ischemia

## Data Availability

The original data presented in the study are openly available on Figshare at https://figshare.com/s/679ee3196be147e80953, accessed on 17 April 2025.

## Abbreviations

AI	Artificial intelligence
ACS	Acute coronary syndrome
ALT	Alanine aminotransferase
AST	Aspartate aminotransferase
AMI	Acute Myocardial Infarction
CA	Cardiac arrest
CI	Confidence intervals
CICU	Cardiac intensive care unit
CS	Cardiogenic shock
CS-Team	Team-based cardiogenic shock
CKD	Chronic kidney disease
DES	Drug eluting stent
DM	Diabetes mellitus
DT	Decision Tree
ECG	Electrocardiographic
ET	Extra Trees
GBC	Gradient Boosting
HTN	Hypertension
ICU	Intensive care unit
IHB	In-hospital bleeding
KNN	K-Nearest Neighbors
LDH	Lactic acid dehydrogenase
LR	Logistic regression
LSTM	Long Short-Term Memory
MCC	Matthews correlation coefficient
MCS	Mechanical circulatory support
ML	Machine learning
NSTEMI	Non-ST-elevation myocardial infarction
NB	Naïve Bayes
OHCA	Out-of-hospital cardiac arrest
P-E-I-CI	Prioritization and Evolving Ischemia in Cardiogenic Instability
PREMs	Patient-reported experience measures
PROMs	Patient-reported outcome measures
QDA	Quadratic Discriminant Analysis
RF	Random Forest
SCAI SHOCK	Society for Cardiovascular Angiography and Interventions Shock
STEMI	ST-elevation myocardial infarction
SVM	Support Vector Machine
VIF	Variance Inflation Factor

## References

[1] Mehta A., Vavilin I., Nguyen A.H., Batchelor W.B., Blumer V., Cilia L., Dewanjee A., Desai M., Desai S.S., Flanagan M.C. (2024). Contemporary approach to cardiogenic shock care: A state-of-the-art review. Front. Cardiovasc. Med..

[2] Laghlam D., Benghanem S., Ortuno S., Bouabdallaoui N., Manzo-Silberman S., Hamzaoui O., Aissaoui N. (2024). Management of cardiogenic shock: A narrative review. Ann. Intensive Care.

[3] Tehrani B.N., Truesdell A.G., Psotka M.A., Rosner C., Singh R., Sinha S.S., Damluji A.A., Batchelor W.B. (2020). A Standardized and Comprehensive Approach to the Management of Cardiogenic Shock. JACC Heart Fail..

[4] Naidu S.S., Baran D.A., Jentzer J.C., Hollenberg S.M., van Diepen S., Basir M.B., Grines C.L., Diercks D.B., Hall S., Kapur N.K. (2022). Standards and Guidelines SCAI SHOCK Stage Classification Expert Consensus Update: A Review and Incorporation of Validation Studies. J. Soc. Cardiovasc. Angiogr. Interv..

[5] Lim H.S., González-Costello J., Belohlavek J., Zweck E., Blumer V., Schrage B., Hanff T.C. (2024). Hemodynamic management of cardiogenic shock in the intensive care unit. J. Heart Lung Transplant..

[6] Pöss J., Desch S., Thiele H. (2014). Shock management in acute myocardial infarction. EuroIntervention.

[7] Elhaddad M., Hamam S. (2024). AI-Driven Clinical Decision Support Systems: An Ongoing Pursuit of Potential. Cureus.

[8] Artificial Intelligence in Healthcare—European Commission. https://health.ec.europa.eu/ehealth-digital-health-and-care/artificial-intelligence-healthcare_en.

[9] Böhm A., Segev A., Jajcay N., Krychtiuk K.A., Tavazzi G., Spartalis M., Kollarova M., Berta I., Jankova J., Guerra F. (2025). Machine learning-based scoring system to predict cardiogenic shock in acute coronary syndrome. Eur. Heart J. Digit. Health.

[10] Stamate E., Piraianu A.-I., Ciobotaru O.R., Crassas R., Duca O., Fulga A., Grigore I., Vintila V., Fulga I., Ciobotaru O.C. (2024). Revolutionizing Cardiology through Artificial Intelligence—Big Data from Proactive Prevention to Precise Diagnostics and Cutting-Edge Treatment—A Comprehensive Review of the Past 5 Years. Diagnostics.

[11] Patrascanu O.S., Tutunaru D., Musat C.L., Dragostin O.M., Fulga A., Nechita L., Ciubara A.B., Piraianu A.I., Stamate E., Poalelungi D.G. (2024). Future Horizons: The Potential Role of Artificial Intelligence in Cardiology. J. Pers. Med..

[12] Piraianu A.-I., Fulga A., Musat C.L., Ciobotaru O.-R., Poalelungi D.G., Stamate E., Ciobotaru O., Fulga I. (2023). Enhancing the Evidence with Algorithms: How Artificial Intelligence Is Transforming Forensic Medicine. Diagnostics.

[13] Alowais S.A., Alghamdi S.S., Alsuhebany N., Alqahtani T., Alshaya A.I., Almohareb S.N., Aldairem A., Alrashed M., Bin Saleh K., Badreldin H.A. (2023). Revolutionizing healthcare: The role of artificial intelligence in clinical practice. BMC Med. Educ..

[14] Totolici G., Miron M., Culea-Florescu A.L. (2024). Automatic Segmentation and Statistical Analysis of the Foveal Avascular Zone. Technologies.

[15] Doolub G., Khurshid S., Theriault-Lauzier P., Lapalme A.N., Tastet O., So D., Langlais E.L., Cobin D., Avram R. (2024). Revolutionising Acute Cardiac Care With Artificial Intelligence: Opportunities and Challenges. Can. J. Cardiol..

[16] Karalis V.D. (2024). The Integration of Artificial Intelligence into Clinical Practice. Appl. Biosci..

[17] Stamate E., Ciobotaru O.R., Arbune M., Piraianu A.I., Duca O.M., Fulga A., Fulga I., Balta A.A.S., Dumitrascu A.G., Ciobotaru O.C. (2024). Multidisciplinary Perspectives of Challenges in Infective Endocarditis Complicated by Septic Embolic-Induced Acute Myocardial Infarction. Antibiotics.

[18] Niculet E., Bobeica C., Stefanopol I.A., Pelin A.M., Nechifor A., Onisor C., Tatu A.L. (2022). Once-Daily Abrocitinib for the Treatment of Moderate-to-Severe Atopic Dermatitis in Adults and Adolescents Aged 12 Years and Over: A Short Review of Current Clinical Perspectives. Ther. Clin. Risk Manag..

[19] Tamis-Holland J.E., Abbott J.D., Al-Azizi K., Barman N., Bortnick A.E., Cohen M.G., Dehghani P., Henry T.D., Latif F., Madjid M. (2024). SCAI Expert Consensus Statement on the Management of Patients With STEMI Referred for Primary PCI. J. Soc. Cardiovasc. Angiogr. Interv..

[20] Q1 Q., So D.Y.F., Boudreau R., Chih S. (2025). The Role of a Cardiogenic Shock Team in Decision Making Surrounding Mechanical Circulatory Support. Can. J. Cardiol..

[21] Recommendations on Digital Interventions for Health System Strengthening. https://www.who.int/publications/i/item/9789241550505.

[22] Johnson K.B., Horn I.B., Horvitz E. (2025). Pursuing Equity With Artificial Intelligence in Health Care. JAMA Health Forum.

[23] Brahmbhatt D.H., Kalra S., Luk A., Billia F. (2025). From Escalate to Elevate: A New Paradigm for Comprehensive Cardiogenic Shock Management. Can. J. Cardiol..

[24] Goh E., Gallo R., Hom J., Strong E., Weng Y., Kerman H., Cool J.A., Kanjee Z., Parsons A.S., Ahuja N. (2024). Large Language Model Influence on Diagnostic Reasoning: A Randomized Clinical Trial. JAMA Netw. Open.

[25] Mebazaa A., Soussi S. (2023). Precision Medicine in Cardiogenic Shock: We Are Almost There!. JACC Heart Fail..

[26] Galusko V., Wenzl F.A., Vandenbriele C., Panoulas V., Lüscher T.F., Gorog D.A. (2025). Current and novel biomarkers in cardiogenic shock. Eur. J. Heart Fail..

[27] Soussi S., Tarvasmäki T., Kimmoun A., Ahmadiankalati M., Azibani F., dos Santos C.C., Duarte K., Gayat E., Jentzer J.C., Harjola V.-P. (2025). Identifying biomarker-driven subphenotypes of cardiogenic shock: Analysis of prospective cohorts and randomized controlled trials. EClinicalMedicine.

[28] Tatu A.L., Nadasdy T., Arbune A., Chioncel V., Bobeica C., Niculet E., Iancu A.V., Dumitru C., Popa V.T., Kluger N. (2022). Interrelationship and Sequencing of Interleukins 4, 13, 31, and 33—An Integrated Systematic Review: Dermatological and Multidisciplinary Perspectives. J. Inflamm. Res..

[29] Byrne R., Coughlan J.J., Rossello X., Ibanez B., Barbato E., Berry C., Chieffo A., Claeys M.J., Dan G.-A., Members of the Task Force for the 2023 ESC Guidelines for the management of acute coronary syndromes (2023). 2023 ESC Guidelines for the management of acute coronary syndromes. Eur. Heart J..

[30] Moreira A., Crispim J. (2024). The importance of the health information systems in value-based healthcare initiatives: A scoping review. Procedia Comput. Sci..

[31] Yuen T., Senaratne J.M. (2024). Definition, Classification, and Management of Primary Noncardiac Causes of Cardiogenic Shock. Can. J. Cardiol..

[32] Arbelo E., Protonotarios A., Gimeno J.R., Arbustini E., Barriales-Villa R., Basso C., Bezzina C.R., Biagini E., Blom N.A., de Boer R.A. (2023). 2023 ESC Guidelines for the management of cardiomyopathies: Developed by the task force on the management of cardiomyopathies of the European Society of Cardiology (ESC). Eur. Heart J..

[33] Bergmark B.A., Mathenge N., Merlini P.A., Lawrence-Wright M.B., Giugliano R.P. (2022). Acute coronary syndromes. Lancet.

[34] Konstantinides S.V., Meyer G., Becattini C., Bueno H., Geersing G.-J., Harjola V.-P., Huisman M.V., Humbert M., Jennings C.S., Jiménez D. (2020). 2019 ESC Guidelines for the diagnosis and management of acute pulmonary embolism developed in collaboration with the European respiratory society (ERS). Eur. Heart J..

[35] Ammirati E., Frigerio M., Adler E.D., Basso C., Birnie D.H., Brambatti M., Friedrich M.G., Klingel K., Lehtonen J., Moslehi J.J. (2020). Management of Acute Myocarditis and Chronic Inflammatory Cardiomyopathy: An Expert Consensus Document. Circ. Heart Fail..

[36] Delgado V., Marsan N.A., de Waha S., Bonaros N., Brida M., Burri H., Caselli S., Doenst T., Ederhy S., Erba P.A. (2023). 2023 ESC Guidelines for the management of endocarditis. Eur. Heart J..

[37] Rudd K.E., Johnson S.C., Agesa K.M., Shackelford K.A., Tsoi D., Kievlan D.R., Colombara D.V., Ikuta K.S., Kissoon N., Finfer S. (2020). Global, regional, and national sepsis incidence and mortality, 1990–2017: Analysis for the Global Burden of Disease Study. Lancet.

[38] De Mello B.H.G., Oliveira G.B.F., Ramos R.F., Lopes B.B.C., Barros C.B.S., de Carvalho E.O., Teixeira F.B.P., Arruda G.D.S., Revelo M.S.C., Piegas L.S. (2014). Validation of the Killip-Kimball Classification and Late Mortality after Acute Myocardial Infarction. Arq. Bras. Cardiol..

[39] ElSayed N.A., Aleppo G., Bannuru R.R., Bruemmer D., Collins B.S., Ekhlaspour L., Gaglia J.L., Hilliard M.E., Johnson E.L., American Diabetes Association Professional Practice Committee (2024). 2. Diagnosis and Classification of Diabetes: Standards of Care in Diabetes—2024. Diabetes Care.

[40] McEvoy J.W., McCarthy C.P., Bruno R.M., Brouwers S., Canavan M.D., Ceconi C., Christodorescu R.M., Daskalopoulou S.S., Ferro C.J., Gerdts E. (2024). 2024 ESC Guidelines for the management of elevated blood pressure and hypertension. Eur. Heart J..

[41] Mach F., Baigent C., Catapano A.L., Koskinas K.C., Casula M., Badimon L., Chapman M.J., De Backer G.G., Delgado V., Ference B.A. (2020). 2019 ESC/EAS Guidelines for the management of dyslipidaemias: Lipid modification to reduce cardiovascular risk: The Task Force for the management of dyslipidaemias of the European Society of Cardiology (ESC) and European Atherosclerosis Society (EAS). Eur. Heart J..

[42] Bai Z., Hu S., Wang Y., Deng W., Gu N., Zhao R., Zhang W., Ma Y., Wang Z., Liu Z. (2021). Development of a machine learning model to predict the risk of late cardiogenic shock in patients with ST-segment elevation myocardial infarction. Ann. Transl. Med..

[43] Okazaki H., Shirakabe A., Hata N., Yamamoto M., Kobayashi N., Shinada T., Tomita K., Tsurumi M., Matsushita M., Yamamoto Y. (2014). New scoring system (APACHE-HF) for predicting adverse outcomes in patients with acute heart failure: Evaluation of the APACHE II and Modified APACHE II scoring systems. J. Cardiol..

[44] Argyriou G., Vrettou C.S., Filippatos G., Sainis G., Nanas S., Routsi C. (2015). Comparative evaluation of Acute Physiology and Chronic Health Evaluation II and Sequential Organ Failure Assessment scoring systems in patients admitted to the cardiac intensive care unit. J. Crit. Care.

[45] Jentzer J.C., Anavekar N.S., Mankad S.V., Khasawneh M., White R.D., Barsness G.W., Rabinstein A.A., Kashani K.B., Pislaru S.V. (2018). Echocardiographic left ventricular diastolic dysfunction predicts hospital mortality after out-of-hospital cardiac arrest. J. Crit. Care.

[46] Youn Y.J., Jeon H.S., Kim Y.I., Lee J., Park Y.J., Cho D., Son J., Ahn M., Ahn S.G., Kim J. (2023). Impact of the ultra-long 48 mm drug-eluting stent on procedural and clinical outcomes in patients with diffuse long coronary artery disease. Clin. Cardiol..

[47] Januszek R.A., Bryniarski L., Siudak Z., Malinowski K.P., Surowiec S., Bryniarski K., Jędrychowska M., Wańha W., Bartuś K., Wojakowski W. (2023). Predictors and trends of contrast use and radiation exposure in a large cohort of patients treated with percutaneous coronary interventions: Chronic total occlusion analysis based on a national registry. Cardiol. J..

[48] Wei Z., Bai J., Dai Q., Wu H., Qiao S., Xu B., Wang L. (2018). The value of shock index in prediction of cardiogenic shock developed during primary percutaneous coronary intervention. BMC Cardiovasc. Disord..

[49] Galván-Román F., Fernández-Herrero I., Ariza-Solé A., Sánchez-Salado J.C., Puerto E., Lorente V., Gómez-Lara J., Martín-Asenjo R., Gómez-Hospital J.A., Comín-Colet J. (2022). Prognosis of cardiogenic shock secondary to culprit left main coronary artery lesion-related myocardial infarction. ESC Heart Fail..

[50] Vahdatpour C., Collins D., Goldberg S. (2019). Cardiogenic Shock. J. Am. Heart Assoc..

[51] Calvão J., Braga M., Brandão M., Campinas A., Alexandre A., Amador A., Costa C., Silva J.C., Silva M., Brochado B. (2023). Acute total occlusion of the unprotected left main coronary artery: Patient characteristics and outcomes. Rev. Port. Cardiol..

[52] Sato T., Saito Y., Suzuki S., Matsumoto T., Yamashita D., Saito K., Wakabayashi S., Kitahara H., Sano K., Kobayashi Y. (2022). Prognostic Factors of In-Hospital Mortality in Patients with Acute Myocardial Infarction Complicated by Cardiogenic Shock. Life.

[53] Donazzan L., Ruzzarin A., Muraglia S., Fabris E., Verdoia M., Zilio F., Caretta G., Pezzato A., Campo G., Unterhuber M. (2025). Predictors and Impact of Cardiogenic Shock in Oldest-Old ST-Elevation Myocardial Infarction Patients. J. Clin. Med..

[54] Sarkar A., Grigg W.S., Lee J.J. (2023). TIMI Grade Flow. StatPearls.

[55] Peters E.J., Bogerd M., Berg S.T., Timmermans M.J.C., Engström A.E., Thiele H., Jung C., Schrage B., Sjauw K.D., Verouden N.J.W. (2024). Characteristics and outcome in cardiogenic shock according to vascular access site for percutaneous coronary intervention. Eur. Heart J. Acute Cardiovasc. Care.

[56] Peters E.J., Kunkel J.B., Bogerd M., Berg S.T., Timmermans M.J.C., Helgestad O.K.L., Ravn H.B., Kraaijeveld A.O., Otterspoor L.C., Sjauw K.D. (2025). Development and validation of a risk score in acute myocardial infarction-related cardiogenic shock. Eur. Heart J. Acute Cardiovasc. Care.

[57] Shen C., Wang S., Huo R., Huang Y., Yang S. (2025). Comparison of machine learning and nomogram to predict 30-day in-hospital mortality in patients with acute myocardial infarction combined with cardiogenic shock: A retrospective study based on the eICU-CRD and MIMIC-IV databases. BMC Cardiovasc. Disord..

[58] Auffret V., Cottin Y., Leurent G., Gilard M., Beer J.C., Zabalawi A., Chagué F., Filippi E., Brunet D., Hacot J.-P. (2018). Predicting the development of in-hospital cardiogenic shock in patients with ST-segment elevation myocardial infarction treated by primary percutaneous coronary intervention: The ORBI risk score. Eur. Heart J..

[59] Jentzer J.C., Burstein B., Van Diepen S., Murphy J., Holmes D.R., Bell M.R., Barsness G.W., Henry T.D., Menon V., Rihal C.S. (2021). Defining Shock and Preshock for Mortality Risk Stratification in Cardiac Intensive Care Unit Patients. Circ. Heart Fail..

[60] Chang Y., Antonescu C., Ravindranath S., Dong J., Lu M., Vicario F., Wondrely L., Thompson P., Swearingen D., Acharya D. (2022). Early Prediction of Cardiogenic Shock Using Machine Learning. Front. Cardiovasc. Med..

[61] Spadafora L., Betti M., D’ascenzo F., De Ferrari G., De Filippo O., Gaudio C., Collet C., Sabouret P., Agostoni P., Zivelonghi C. (2025). Impact of In-Hospital Bleeding on Post-Discharge Therapies and Prognosis in Acute Coronary Syndromes. J. Cardiovasc. Pharmacol..

